# Negative feedback loop between p66^Shc^ and ZEB1 regulates fibrotic EMT response in lung cancer cells

**DOI:** 10.1038/cddis.2015.74

**Published:** 2015-04-02

**Authors:** X Li, D Gao, H Wang, X Li, J Yang, X Yan, Z Liu, Z Ma

**Affiliations:** 1 Department of Biochemistry and Molecular Biology, Immunology, School of Basic Medical Sciences, 2011 Collaborative Innovation Center of Tianjin for Medical Epigenetics, Tianjin Key Laboratory of Medical Epigenetics, Tianjin Medical University, Tianjin 300070, China; 2 Laboratory of Epigenetics and Tumorigenesis, Tianjin Research Center of Basic Medical Sciences, Tianjin Medical University, Tianjin 300070, China; 3 Department of Pharmaceutical Engineering, Key Laboratory of Systems Bioengineering (Ministry of Education), School of Chemical Engineering and Technology, Tianjin University, 92 Weijin Road, Nankai District, Tianjin 300072, China; 4 Key Laboratory of Immune Microenvironment and Disease (Ministry of Education), Tianjin Medical University, Tianjin 300070, China; 5 Key Laboratory of Hormones and Development (Ministry of Health), Metabolic Diseases Hospital, Tianjin Institute of Endocrinology, Tianjin Medical University, Tianjin 300070, China

## Abstract

The epithelial-to-mesenchymal transition (EMT) program is crucial for the epithelial cancer progression and fibrotic diseases. Our previous work has demonstrated that p66^Shc^, a focal adhesion-associated adaptor protein, is frequently downregulated in lung cancers and its depletion promotes metastasis behavior through anoikis resistance. However, mechanism underlying loss of p66^Shc^ and EMT response is not fully understood. Here, we showed that p66^Shc^ deficiency enhanced the expression of ZEB1, the known mesenchymal transcription factor and consequently increased Vimentin, and decreased epithelial markers of E-cadherin and *β*-catenin. p66^Shc^ depletion also increased cell invasion and migration. In addition, ChIP and luciferase assays showed that these effects were directly mediated by ZEB1 repression of p66^Shc^ promoter. Thus, our findings define a critical role of p66^Shc^ in the suppression of fibrotic EMT response with a negative feedback loop between p66^Shc^ and ZEB1 in lung epithelial cancer cells.

Owing to its high metastasis, lung cancer is clinically one of the leading causes of cancer-related death worldwide.^1^ Although the exact mechanisms are not well understood, the process of oncogenic epithelial-to-mesenchymal transition (EMT) seems to be involved in these neoplastic processes.^2, 3, 4^ Oncogenic EMT is a developmental program implying a substantial chromatin plasticity and change in gene expression profile for cell reprogramming,^5^ and also is an improper occurrence and is associated with the acquisition of a new phenotype, which seems crucial for tumor progression.^6^ This process involves loss of cell–cell adhesion and increased cellular mobility,^7^ as well as anoikis (detachment-induced cell death) resistance by constitutively activating specific pro-survival signal pathways.^8, 9, 10, 11, 12, 13^ Most of these changes are related to the switch-off or decreased expression of epithelial genes in contrast with a new expression or overexpression of mesenchimal genes and markers, presumably linked to the precise temporal and spatial requirement of EMT in specific cell types at precisely coordinated times during development.^14^ Oncogenic EMT induction involves a complex network of signaling molecules and transcription factors, including SNAIL, zinc-finger E-box-binding homeobox 1 (ZEB1) and basic helix-loop-helix transcription factors, as well as a diverse array of cytokines and growth factors including transforming growth factor*-β* (TGF*β*).^6, 15^ Currently, targeting EMT process remains a very likely candidate for carcinoma metastasis, with opportunities for diagnosis, prognosis, and treatment to be explored.^6^ This simplified thought integrates clinically relevant aspects of dormancy, metastatic tendency and therapy resistance.^11^

The mammal adaptor proteins Shc1 (p66^Shc^, p52^Shc^ and p46^Shc^) share a C-terminal Src homology 2 domain, a central collagen-homologous (CH1) domain and an N-terminal phosphotyrosine-binding domain and have N termini of different lengths.^16^ Despite their high structural similarity, p66^Shc^ and p52^Shc^/p46^Shc^ are involved in multiple signaling mechanisms to support diversity in cell homeostasis.^17^ Of the two Shc1 transcripts and three isoforms, p66^Shc^ activates pro-apoptosis in response to oxidative stress and functions as a critical regulator of longevity in mice.^18, 19^ Structurally, an additional 110-amino-acid CH2 domain of p66^Shc^ at the amino terminal differs from p52^Shc^ and p46^Shc^ isoforms.^18^ We have previously shown that p66^Shc^ is also a focal adhesion-associated protein, permits anchorage sensing through a RhoA-dependent mechanosensory test of the environment, mediates anoikis in epithelial cells, and thus has an important role in preventing solid cancer metastasis.^20^ In addition, a stress related transcription factor Nrf2 binds to p66^Shc^ promoter and promotes p66^Shc^ transcription, which links to reactive oxygen species (ROS) control and tumorigenesis.^19, 21^ Loss of p66^Shc^ expression leads to unrestrained k-Ras activation and anoikis resistance, allowing anchorage independence *in vitro* and aggressive metastasis of lung cancer cells *in vivo*.^22, 23^ Anoikis resistance is one of the hallmarks of oncogenic EMT,^24, 25^ and ZEB1 is required for the neurotrophic tyrosine kinase receptor, TrkB-induced EMT, anoikis resistance and metastasis.^26^ Similarly, p66^Shc^ depletion contributes to anoikis resistance and promotes metastasis in human lung cancer cells.^22^ Therefore, it is possible that aberrant expression of p66^Shc^ may lead to the activation of a developmentally regulated fibrotic EMT response in lung cancer cells.

We report here that metastasis suppressor p66^Shc^ expression is dependent on cell density in lung cancer cells and represses the oncogenic EMT-activator ZEB1. During oncogenic EMT, p66^Shc^ is shown to be downregulated, promoting ZEB1 activity, downregulating E-cadherin and *β*-catenin. Considering the role of p66^Shc^ in metastasis suppression, we propose that a novel feedback loop between ZEB1 and p66^Shc^ mediates fibrotic EMT response.

## Results

### TGF*β*1 induces p66^Shc^ downregulation

**Figure 1:**
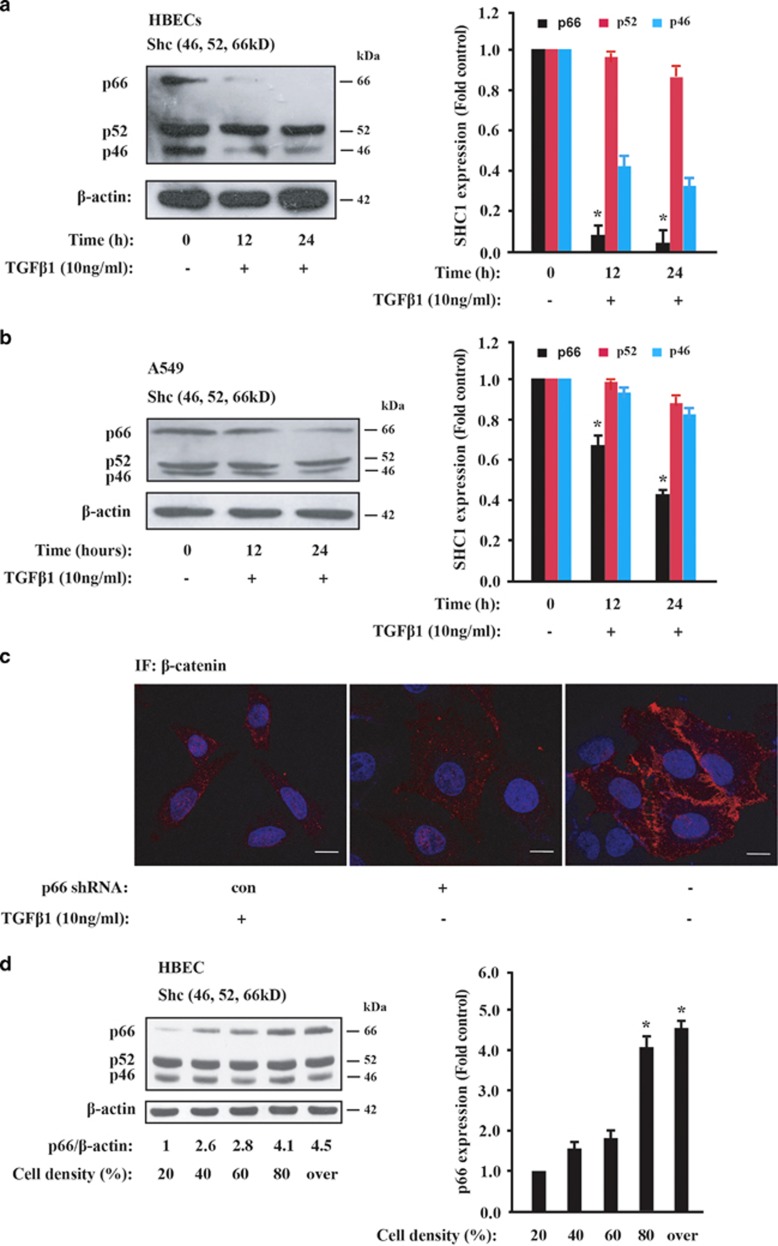
p66^Shc^ progressively decreases by TGF*β*1 treatment. (**a**, **b**) Immunoblot analysis of Shc1 protein level in HBECs and A549 cells in the presence of 10 ng/ml TGF*β*1 at the indicated time, respectively, with *β*-actin as a loading control. Results shown are representative of three independent blots (left panel). Graph depicts densitometric analysis of p66, p52 and p46 band intensity, normalized to *β*-actin levels and expressed as fold change from untreated control (right panel). *P<0.05 as compared with the untreated control. (**c**) A549 cells were infected with the control shRNA against luciferase or no treatment (left and right panels, respectively) or p66^Shc^ shRNA (middle panel) and imaged with confocal microscopy. Immunofluorescence staining for *β*-catenin (red) and DAPI counterstaining for DNA (blue). Ten  ng/ml TGF*β*1 treatment for 16 h served as a positive control (left panel). Scale bars, 5 *μ*m. (**d**) Immunoblot analysis of Shc1 in HBEC cells at the indicated cell density. Quantification of p66^Shc^ protein expression relatively to the constitutive *β*-actin expression at the indicated levels of cell confluence are shown in the right panel. Results shown are representative of three independent blots. *P<0.01 as compared with the cell density of 20%

TGF*β*1 has been traditionally used to investigate the EMT process and fibrotic response in epithelial cancer cells.^2, 27, 28, 29^ As shown in Figures 1a and b, p66^Shc^ protein level was significantly decreased by time-dependent TGF*β*1 treatment in human bronchial epithelial cells (HBECs) and human lung adenocarcinoma A549 cells, respectively. Also, similar tendency was observed in TGF*β*1-treated hepatocellular cancer HepG2 cells (Supplementary Figure 1). These data indicate that p66^Shc^ is endogenously regulated for epithelial maintenance and inhibits the TGF*β*1-induced fibrotic EMT process.

To investigate the function of p66^Shc^ in fibrotic EMT response, we used short hairpin RNA (shRNA) specifically against p66^Shc^ and created stable p66^Shc^ knockdown lung cancer A549 cells as performed previously.^30^ Immunofluorescence staining of *β*-catenin showed that knockdown of p66^Shc^ resulted in a phenotypic change from a ‘cobble-stone'-like shape to a fibroblastic shape (Figure 1c, middle panel). A similar cell morphology change with TGF*β*1 treatment was observed in Figure 1c, left panel, when compared with the non-treated control cells (Figure 1c, right panel). *β*-catenin immunostaining showed its dissociation from plasma and its relocalization to the cytosol and nucleus in p66^Shc^-depleted cells (Figure 1c, left panel), confirming that p66^Shc^ was involved in fibrotic EMT response with cell morphological change.

The mammal *SHC1* locus contains two alternative promoters, which drive the constitutive transcription of p52/p46^Shc^ and the differential expression of p66^Shc^ by epigenetic modification, respectively.^16, 23^ Previously, we reported that p66^Shc^ but not p52^Shc^ expression is increased with cell density in cultured mouse fibroblast NIH 3T3 and MDCK epithelial cells.^20^ We hypothesized that p66^Shc^ may also be regulated by cell density in epithelial lung cancer cells. To test this possibility, we cultured HBECs and A549 cells with different cell density and determined p66^Shc^ expression by western blot. Indeed, p66^Shc^ protein level was nearly four fold induction under over confluence when compared to sparse conditions (Figure 1d; Supplementary Figure 2a). Similar results were obtained in neuroendocrine H1155 cells (Supplementary Figure 2b). These findings indicate that the expression level of p66^Shc^ positively correlates with cell density, which might be closely connected to the cell–cell contact and fibrotic EMT phenotype.

### p66^Shc^ depletion confers the EMT-like phenotype

**Figure 2:**
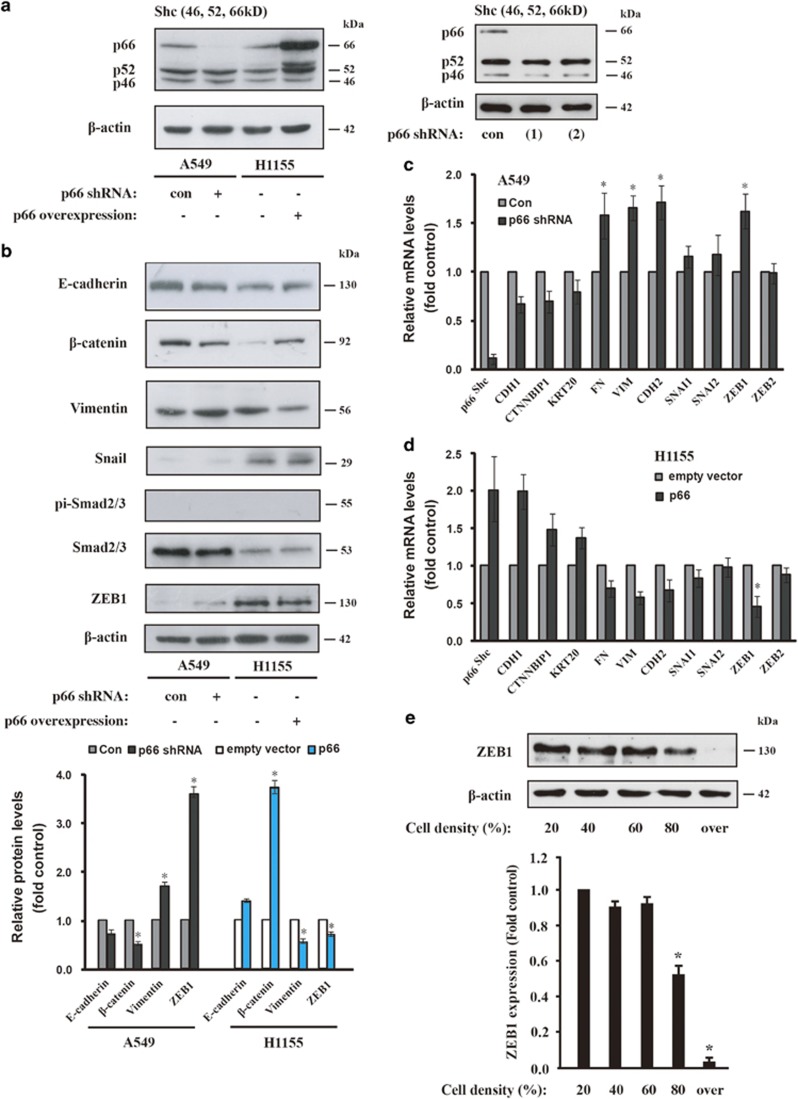
p66^Shc^ depletion confers EMT-like phenotype. (**a**) Immunoblot analysis of Shc1 expression in A549 cells expressing p66^Shc^ shRNA and in H1155 cells overexpressing p66^Shc^, respectively. Right panel, western blotting shows knockdown effect by shRNA (1) and (2) for p66^Shc^ as well as control (con) against fire fly luciferase in A549 cells. (**b**) Whole-cell lysates were subjected to an immunoblot analysis with antibodies to E-cadherin, *β*-catenin, Vimentin, ZEB1, Snail, Smad2/3, phosphor-Smad2/3 and *β*-actin. Graph depicts densitometric analysis of protein intensity as indicated in the upper panel, normalized to *β*-actin levels and expressed as fold change from untreated control (lower panel). *P<0.05 as compared with the untreated control. (**c**) Expression of EMT markers was validated via quantitative RT-PCR (qRT-PCR) in A549 cells expressing p66^Shc^ shRNA(1) and (**d**) in H1155 cells overexpressing p66^Shc^, respectively. Normalized signals to GAPDH represent mean±S.D. of triplicates. *P<0.05. (**e**) Immunoblot analysis of ZEB1 in H1155 cells at the indicated cell density. Quantification of ZEB1 protein expression are shown in the lower panel as performed in Figure 1d. *P<0.01 as compared with the cell density of 20%

Given that p66^Shc^ expression is necessary for maintaining the epithelial phonotype, we then asked whether loss of p66^Shc^ in cancer cells is related to the EMT-like process. We conducted lentiviral shRNA knockdown of p66^Shc^ to attenuate its expression in A549 cells and lentiviral overexpression in H1155 cells relative to the empty vector control (Figure 2a). Depletion of p66^Shc^ decreased the protein level of E-cadherin and *β*-catenin and markedly increased the level of the mesenchymal marker Vimentin (Figure 2b). Accordingly, p66^Shc^ overexpression in H1155 cells showed the opposite tendency compared with the empty vector control (Figure 2b). Exposure of A549 cells to TGF*β*1 also decreased the epithelial marker E-cadherin, whereas phosphor-Smad2/3, a downstream target of TGF*β*1 pathway increased (Supplementary Figure 3). However, TGF*β*1-activated phosphor-Smad2/3 was not activated by p66^Shc^ depletion in A549 cells or p66^Shc^ overexpression in H1155 cells (Figure 2b; Supplementary Figure 3), which indicates its unique role of p66^Shc^ in the EMT process. Significantly, loss of p66^Shc^ upregulated ZEB1 at both the mRNA and protein levels (Figures 2b and d) and Vimentin (VIM) and Fibronectin (FN) at the mRNA level as well (Figures 2c and d). However, ZEB1 protein level decreased and p66^Shc^ protein level increased under overconfluence of cell density when compared with sparse conditions in H1155 cells (Figure 2e; Supplementary Figure 2b). The expression at the mRNA and protein levels of other transcriptional repressors Snail1 and Snail2 (also known as SLUG) was not significantly altered by manipulated p66^Shc^ expression in A549 and H1155 cells, respectively (Figures 2b and d), which is consistent with a unique role for p66^Shc^ in maintaining epithelial integrity. Collectively, these data indicated that the loss of p66^Shc^ promotes fibrotic EMT response in lung cancer cells.

### p66^Shc^ expression is negatively regulated by ZEB1

**Figure 3:**
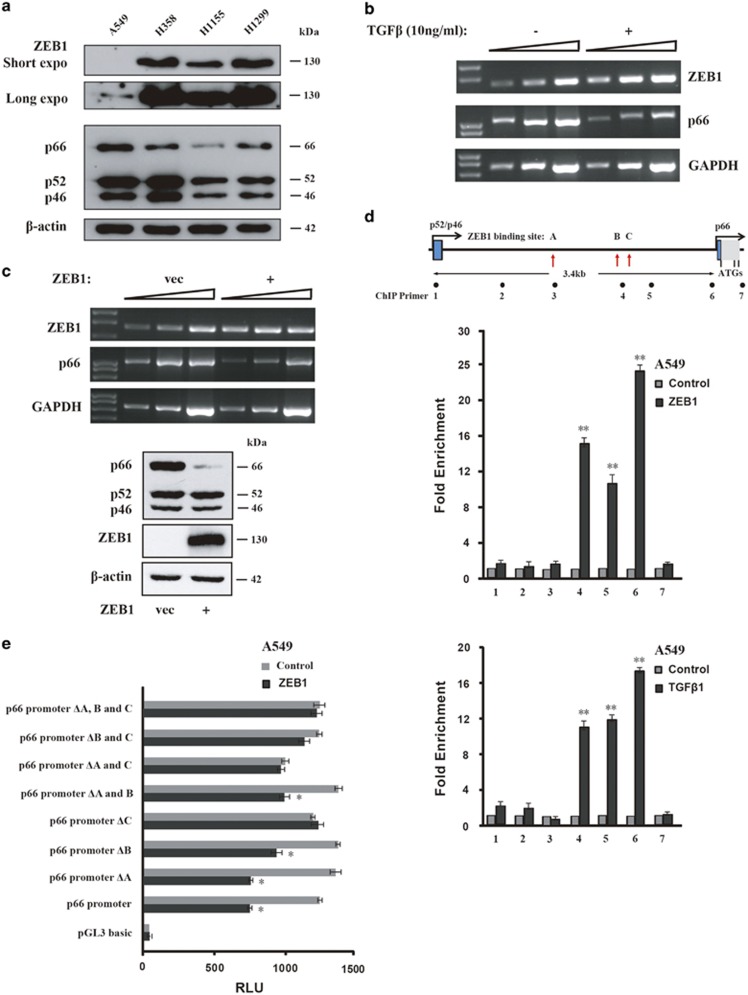
ZEB1 directly suppresses p66^Shc^ expression. (**a**) Immunoblot analysis of ZEB1 (upper two panels showing short and long exposure time, respectively) and Shc1 expression in various lung cancer cell lines as indicated. (**b**) A549 cells were treated with TGF*β* (10ng/ml) for 12 h, and mRNA levels of ZEB1 and p66^Shc^ were determined on RT-PCR with a threefold serial dilution series of the templates. The housekeeping gene *GAPDH* transcripts were used as internal controls. (**c**) Upper panel, RT-PCR analysis as in **b** after ZEB1 overexpression. Lower panel, immunoblot analysis with indicated antibodies. (**d**) ChIP assay was performed on A549 cells transfected with ZEB1 (upper panel) or TGF*β*1 treatment for 16 h (lower panel). The precipitated chromatin was PCR-amplified with the use of specific primers in the p66^Shc^ promoter (Supplementary Table 1) as indicated by black dots. Vertical red arrows indicate three ZEB1-binding sites, namely A, B and C, respectively (see Materials and methods). Bar graphs show fold enrichment of ZEB1 binding. Data represent the mean±S.E.M. of three experiments. **P<0.01 as compared with the mouse IgG control. (**e**) Repressor activity of ZEB1 on p66^Shc^ promoter in A549 cells. ZEB1 expression plasmid was cotransfected with luciferase reporter containing wild type or p66^Shc^ promoter with ZEB1-binding site deletion (ΔA, B and C, respectively). Activity (relative luminescence unit, RLU) was normalized to that present in cells transfected with control empty vector. Mean±S.E.M. of three determinations is shown. *P<0.05 as compared with the empty vector

As the expression level showed the opposite correlation between ZEB1 and p66^Shc^, we assessed whether transcription factor ZEB1 also directly regulates p66^Shc^ transcription. Then, we examined the protein level of ZEB1 in different lung cancer cell lines and we found weak ZEB1 was detectable in A549 cells when given a longer exposure compared with a visible band in H1155 cells (Figure 3a), which is consistent with the previous report showing endogenous ZEB1 expression in both A549 and H1155 cells.^31^ Less p66^Shc^ protein level was at least in part related to higher ZEB1 expression level (Figure 3a). In addition, TGF*β*1 treatment or ZEB1 overexpression significantly suppressed p66^Shc^ at the mRNA and protein levels, respectively (Figures 3b and c). We also confirmed that overexpression of ZEB1 bound to the expected ZEB1-binding sites of p66^Shc^ promoter, and TGF*β*1 treatment also induced the similar ZEB1-binding tendency in A549 cells (Figure 3d). Luciferase assay also showed that depletion of ZEB1-binding sites decreased p66^Shc^ promoter activity in ZEB1-overexpressing A549 cells (Figure 3e). Taken together, these data suggest that p66^Shc^ depletion promotes the EMT-like phenotype, which is at least partially mediated by elevated ZEB1 transcriptional regulation.

### p66^Shc^ depletion increases lung cancer cell invasion and migration

**Figure 4:**
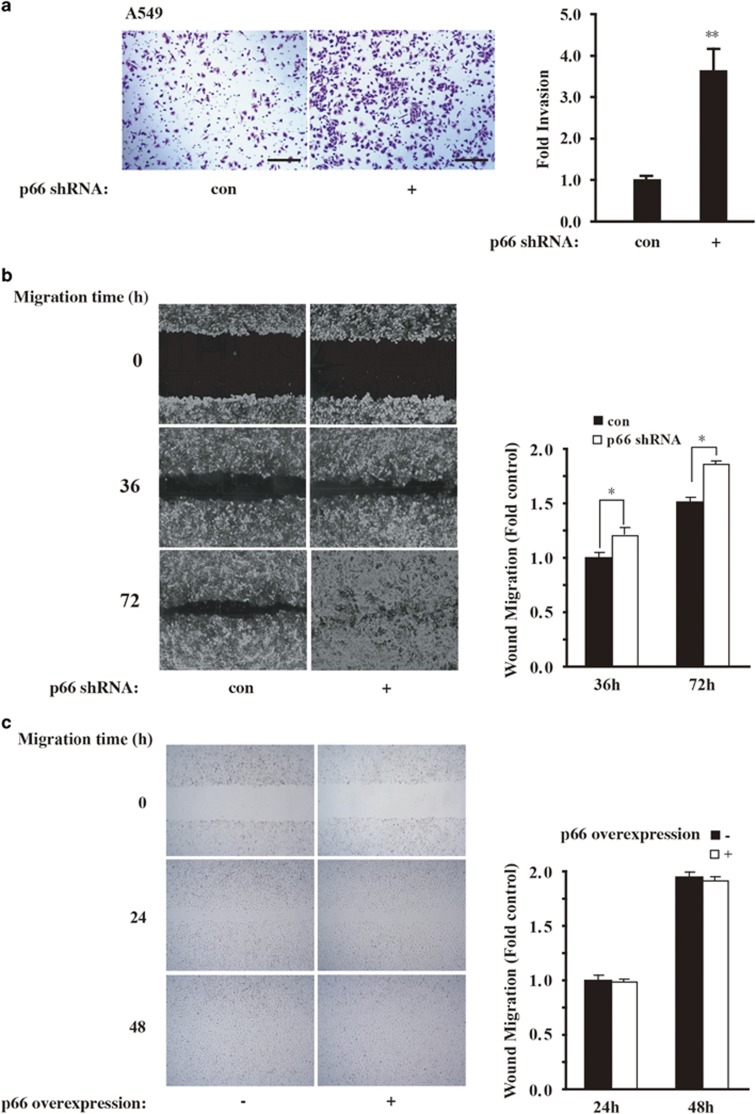
p66^Shc^ depletion increases cell invasion and migration. (**a**) Boyden chamber assay for A549 cells transduced with control or p66^Shc^ shRNA as described in Materials and methods. Scale bars, 100 *μ*m. Quantification of invasion change are shown in the right panel. **P<0.01 as compared with the cells treated with control shRNA alone. (**b**) A549 cells were transduced as in **a** and plated on fibronectin-coated coverslips. Phase-contrast images were obtained immediately after wounding and at 36 and 72 h after wounding. Quantification of mean speed of cells migrating into the wound shown in the right panel. Error bars represent S.E.M. *P<0.05 as compared with the cells treated with control shRNA alone, based on three independent experiments. (**c**) A549 p66^Shc^-overexpressing cells were plated on fibronectin-coated coverslips. Phase-contrast images were obtained immediately after wounding and at 24 and 48 h after wounding as in **b**, respectively

EMT response is well characterized by the loss of cell–cell adhesions and apicobasal polarity, and the transition to a cell type with a more spindle-like morphology that is capable of invading the extracellular matrix (ECM).^32, 33^ Now that p66^Shc^ differentially regulates the expression of EMT-related markers, we inquired whether loss of p66^Shc^ may modulate cellular motility. As expected, knockdown of p66^Shc^ in A549 cells increased invasion ability (Figure 4a) as well as migratory potential (Figure 4b). However, overexpression of p66^Shc^ in A549 cells did not show reverse tendency (Figure 4c). On the other hand, p66^Shc^ overexpression in H1155 cells resulted in mitigated cell invasion (Supplementary Figure 4). When combined, these data indicate that loss of p66^Shc^ in lung cancer cells may increase their malignance with increased cell invasion and migration.

## Discussion

Here we report the mechanisms of the p66^Shc^-maintained epithelial phenotype and demonstrate that depletion of p66^Shc^ induces fibrotic EMT response through ZEB1 activation in lung cancer cells. Significantly, p66^Shc^ overexpression has a critical role in the establishment of epithelial morphogenesis through upregulating the expression of proteins that are responsible for the formation of cell–cell contacts and cell adhesion.^23^ In all of these properties, p66^Shc^ appears to function as an important suppressor for fibrotic EMT response and its suppressive potential of metastasis in solid tumors.

Epithelial plasticity responses depend on a spectrum of intrinsic and/or extrinsic signaling changes and transitions including cell density.^34^ Two principle types of metastatic progression have been proposed that are phenotypic plasticity involving transient EMT–MET processes and intrinsic genetic alterations keeping cells in an EMT and stemness state.^11, 35^ During oncogenic EMT, polarity proteins fail to localize correctly to the membrane, promoting TGF*β* signaling, which, in turn, stabilizes the EMT phenotype and the consequent resistance to anoikis.^24, 36^ The EMT-activator ZEB1, a well-characterized EMT transcription factor, is a crucial activator of tumorigenicity and metastasis.^11, 37, 38^ ZEB1 also mediates non-cancer stem cell to cancer stem cell (CSC) conversion, which is consistent with the idea that the EMT generates cells with CSC-like activity.^39, 40^

The observation that p66^Shc^ depletion directly upregulates expression of ZEB1 in A549 cells raises the possibility that p66^Shc^ may function as an upstream or ‘master' regulator of fibrotic EMT response. In this context, it is interesting that p66^Shc^ rather than the other two Shc1 isoforms (Figures 1a and b) and ZEB1 are all involved in the EMT process, whereas ZEB1 mediates cell fate determination in solid cancer cells. Therefore, p66^Shc^ and ZEB1 may integrate different environmental cues to guide and maintain the metastatic behavior. ZEB1 is induced in response to TGF*β* and WNT proteins as well as TWIST1 cooperatively with SNAIL1 (also known as SNAIL), promoting EMT.^6^ Increased ROS induce miR-200c and other miR-200 family members, which targets ZEB1 downregulation; however, in p66^Shc^ knockout mice skeletal muscle cells, upregulation of miR-200c was inhibited.^41^ Furthermore, increased cellular p66^Shc^ results in further increases in ROS accumulation.^42^ Therefore, the integrative role proposed for p66^Shc^ in normal epithelial makes it a particularly interesting candidate for a role in tumorigenesis by mediating fibrotic EMT response and its targets regulation. Consistent with this scenario, downregualted p66^Shc^ may increase cancerous stemness as well as fibrotic EMT response in given solid cancer cells. Thus, a negative feedback of p66^Shc^ and ZEB1 may exist during fibrotic EMT response where p66^Shc^ has an important role of inside-out signaling of mechanosensory test of anchorage.

Differentiated epithelial and endothelial cells sense their location through specific interactions with the ECM as well as neighboring cells, presumably to prevent cellular vagrancy and consequent tissue disorgamization.^43^ Protection from detachment-induced cell death, termed anoikis, facilitates metastasis, which is responsible for most of the clinical patients' death.^44, 45^ Anoikis resistance, the loss of anoikis sensitivity, was reported to accompany EMT,^9, 10^ and the adaptor protein p66^Shc^ has been showed to mediate anoikis through Rho A activation.^20^ p66^Shc^ expression is limited to the epithelial and endothelial cells but not the lymphatic cells that survive anchorage independence.^18^ Although anoikis is largely attributed to the loss of integrin-related ‘outside-in' survival signals, our previous work demonstrates a novel ‘inside-out' attachment sensing role for the adapter protein p66^Shc^ in promoting anoikis and suppressing metastasis via Ras-dependent control of proliferation and survival.^22, 46^ In solid tumor cells, however, the promoter of p66^Shc^ is hypermethylated and consequently silenced its expression, which may contribute to the invasion and metastasis cascade.^16, 21^ Furthermore, lung cancer cells survive anchorage deprivation through repression of p66^Shc^ and other genes by co-option of Aiolos, a hematopoietic transcription factor, which is required for lymphopoiesis.^23^ We have also shown that p66^Shc^ suppression results in cell cycle arrest in normal epithelial cells secondary to the activation of the tumor suppressor pRB.^22^ pRB inactivation contributes to deregulation of E-cadherin and induction of cellular phenotypic changes that are characteristic of the EMT, as well as loss of cell proliferation control.^47^ Previously, we also observed variability of p66^Shc^ downregulation in primary human lung cancer tissues.^21, 23^ This may represent a correlation with the EMT–MET process and the various expression levels of p66^Shc^ in human lung tumor tissues.^23^ Thus, p66^Shc^ may inhibit fibrotic EMT response and mediate anoikis and consequently suppresses metastasis.

In summary, we have demonstrated that p66^Shc^-maintained epithelial phenotype and demonstrate that p66^Shc^ depletion induces fibrotic EMT response through ZEB1 activation in lung cancer cells. During this response, ZEB1 associates with p66^Shc^ promoter and suppresses p66^Shc^ transcription and promotes cancer cells metastasis by bypassing anoikis. On the other hand, depletion of p66^Shc^ in metastatic cancer cells increases the expression of ZEB1, leading to the reduced p66^Shc^ transcription level and disruption of cell–cell contacts. Thus, p66^Shc^ inhibits fibrotic EMT response and mediates anoikis and consequently suppresses metastasis. For these reasons, additional analyses of the mechanism of p66^Shc^ reactivation and its consequences of metastatic inhibition are warranted.

## Materials and Methods

### Reagents and immunoblot

TGF*β*1 was purchased from Thermo Fisher Scientific, Waltham, MA, USA. Cells were lysed in RIPA lysis buffer containing 20 mM Tris, pH 8.0, 150 mM NaCl, 10 mM NaF, 0.1% SDS, 1% Nonidet P-40, 1 × protease inhibitor mixture (Roche, Penzberg, Germany). Blots were performed for E-cadherin, *β*-catenin, Vimentin (BD Biosciences, San Jose, CA, USA), ZEB1, Snail (Abcam, Cambridge, MA, USA), phospho-Smad2/3 (Ser465/467) (Cell Signaling Technology, Danvers, MA, USA), Shc1 (BD Biosciences) or horseradish peroxidase-conjugated secondary antibodies (Bio-Rad, Hercules, CA, USA), followed by ECL detection reagents (GE Healthcare, Pittsburgh, PA, USA). Blots were stripped and reprobed for total Smad2/3 (Cell Signaling Technology) or *β-*actin (Chemicon, Darmstadt, Germany).

### Cell culture and viral infection

HBECs were immortalized by overexpression of cyclin-dependent kinase 4 and human telomerase reverse transcriptase and were cultured in keratinocyte serum-free medium (Invitrogen, Karlsruhe, Germany) with 5 ng/ml epidermal growth factor and 50 *μ*g/ml bovine pituitary extract.^48^ Phoenix-293, HepG2, H358, H1155, H1299 and A549 cells were obtained from American Type Culture Collection (Manassas, VA, USA). For lentiviral transduction, phoenix-293 cells were cotransfected with the transfer constructs and the third-generation packaging plasmids pMD2.VSVG, pMDLg/pRRE, and pRSV-REV, and fresh supernatant was used for infection. After 8 h infection, cells were washed and allowed to recover for 24 h prior to further procedure. Targeting sequences for human p66^Shc^ are listed in Supplementary Table 1.

### ChIP assay

Chromatin immunoprecipitation (ChIP) assay was performed as previously described.^49^ Briefly, cells were cross-linked with 1% formaldehyde for 10 min, quenched with 0.125 M glycine, and lysed on ice for 10 min in cell-swelling buffer containing 5 mM Pipes (pH 8.0), 85 mM KCl, 0.5% NP-40, 0.5 mM phenylmethylsulfonyl fluoride, and 100 ng/ml leupeptin and aprotinin. Nuclei were collected and resuspended in sonication buffer with 1% SDS, 10 mM EDTA, 50 mM Tris-HCl (pH 8.1), 0.5 mM phenylmethylsulfonyl fluoride, and 100 ng/ml leupeptin and aprotinin and incubated on ice for 10 min. Samples were sonicated to an average length of 0.5 kb and antibodies against ZEB1 (Millipore, Darmstadt, Germany), mouse normal IgG (Santa Cruz, Santa Cruz, CA, USA) were added to each aliquot of chromatin and incubated on a rotating platform overnight at 4 °C. Antibody–protein–DNA complexes were isolated by immunoprecipitation with protein A agarose beads. Following extensive washing, bound DNA fragments were eluted and analyzed by subsequent PCR. The putative ZEB1-binding sites from ENCODE were indicated as site A(−1961 to −1955, relative to the transcription start site of p66^Shc^), B(−1378 to −1373) and C(−1285 to −1243), respectively. Primer sequences are listed in Supplementary Table 1.

### Luciferase assay

A549 cells grown in 24-well plates were cotransfected with 100 ng of reporter construct, 50 ng of expression vector, and 5 ng of internal control Renilla construct (Promega, Madison, WI, USA) using Lipofectamine 2000 (Invitrogen). Thirty-six hours after transfection, luciferase activity was monitored using the Dual-Luciferase Reporter Assay System and a luminometer (Promega).

### Transwell invasion and boyden chamber cell migration assay

Cells (1 × 10^5^) were plated without serum on 8-*μ*m pore size Transwell filters (Corning, Corning, NY, USA) in the Matrigel invasion assay as described.^23^ The Boyden chamber cell migration assay was performed in Boyden chambers without Matrigel as described.^31^ Assays were stained and quantified after cells migrated for 48 h.

### Wound-healing assay

Migration into wounds was examined on fibronectin-coated coverslips. At least five wounded fields per coverslip were analyzed on six coverslips per condition, and identical fields were photographed under phase at 0, 36, and 72 h.

### Real-time qRT-PCR

RNA was isolated using RNeasy Mini kit (Qiagen, Hilden, Germany) and used for real-time quantitative reverse transcription (qRT)-PCR using SYBR Green in an ABI PRISM 7500 sequence detection system with a 96-block module and automation accessory (Applied Biosystem, Karlsruhe, Germany). *Glyceraldehyde 3-phosphate dehydrogenase* was used as an internal control gene. All samples were analyzed in triplicate. The primer sequences are listed in Supplementary Table 1.

### Immunofluorescence analysis

Cells were plated on 35-mm coverslip-bottom dishes coated with fibronectin. Cells were washed with PBS, fixed with 4% formaldehyde for 15 min, permeabilized with 0.5% Triton X-100 for 10 min, blocked with 10% goat serum for 1 h, incubated with proper primary antibodies for 1 h, with secondary antibodies conjugated with fluorescence dye (Alexa Fluor 555, Invitrogen) for 1 h, and counterstained with 4′,6-diamidino-2-phenylindole for 10 min. At least 50 cells from more than 10 fields were counted for statistical analysis.

### Statistical analysis

Data are expressed as mean±S.E.M. from at least three independent experiments. Statistical analysis was performed with ANOVA for multiple variables and with *t*-tests for comparison of two groups with normal distribution.

## Abbreviations

ChIP: chromosomal immunoprecipitation
CSC: cancer stem cell
ECM: extracellular matrix
EMT: epithelial-to-mesenchymal transition
HBEC: human bronchial epithelial cell
MET: mesenchymal-to-epithelial transition
ROS: reactive oxygen species
RT-PCR: reverse transcription-PCR
shRNA: short hairpin RNA
TGF*β*1: Transforming growth factor-*β*1
ZEB1: zinc-finger E-box-binding homeobox 1

## References

[bib1] 1Zimmermann S, Dziadziuszko R, Peters S. Indications and limitations of chemotherapy and targeted agents in non-small cell lung cancer brain metastases. Cancer Treat Rev 2014; 40: 716–722. 24759599 10.1016/j.ctrv.2014.03.005

[bib2] 2De Craene B, Berx G. Regulatory networks defining EMT during cancer initiation and progression. Nat Rev Cancer 2013; 13: 97–110. 23344542 10.1038/nrc3447

[bib3] 3Guo W, Giancotti FG. Integrin signalling during tumour progression. Nat Rev Mol Cell Biol 2004; 5: 816–826. 15459662 10.1038/nrm1490

[bib4] 4Geiger TR, Peeper DS. Metastasis mechanisms. Biochim Biophys Acta 2009; 1796: 293–308. 19683560 10.1016/j.bbcan.2009.07.006

[bib5] 5Davis FM, Stewart TA, Thompson EW, Monteith GR. Targeting EMT in cancer: opportunities for pharmacological intervention. Trends Pharmacol Sci 2014; 35: 479–488. 25042456 10.1016/j.tips.2014.06.006

[bib6] 6Lamouille S, Xu J, Derynck R. Molecular mechanisms of epithelial-mesenchymal transition. Nat Rev Mol Cell Biol 2014; 15: 178–196. 24556840 10.1038/nrm3758 PMC4240281

[bib7] 7Kalluri R, Weinberg RA. The basics of epithelial-mesenchymal transition. J Clin Invest 2009; 119: 1420–1428. 19487818 10.1172/JCI39104 PMC2689101

[bib8] 8Meredith JE Jr, Fazeli B, Schwartz MA. The extracellular matrix as a cell survival factor. Mol Biol Cell 1993; 4: 953–961. 8257797 10.1091/mbc.4.9.953 PMC275725

[bib9] 9Thompson EW, Newgreen DF, Tarin D. Carcinoma invasion and metastasis: a role for epithelial-mesenchymal transition? Cancer Res 2005; 65: 5991–5995 discussion 5995. 16024595 10.1158/0008-5472.CAN-05-0616

[bib10] 10Kumar S, Park SH, Cieply B, Schupp J, Killiam E, Zhang F et al. A pathway for the control of anoikis sensitivity by E-cadherin and epithelial-to-mesenchymal transition. Mol Cell Biol 2011; 31: 4036–4051. 21746881 10.1128/MCB.01342-10 PMC3187352

[bib11] 11Brabletz T. To differentiate or not—routes towards metastasis. Nat Rev Cancer 2012; 12: 425–436. 22576165 10.1038/nrc3265

[bib12] 12Frisch SM, Francis H. Disruption of epithelial cell-matrix interactions induces apoptosis. J Cell Biol 1994; 124: 619–626. 8106557 10.1083/jcb.124.4.619 PMC2119917

[bib13] 13Gilmore AP. Anoikis. Cell Death Differ 2005; 12: 1473–1477. 16247493 10.1038/sj.cdd.4401723

[bib14] 14Katsuno Y, Lamouille S, Derynck R. TGF-beta signaling and epithelial-mesenchymal transition in cancer progression. Curr Opin Oncol 2013; 25: 76–84. 23197193 10.1097/CCO.0b013e32835b6371

[bib15] 15Singh A, Settleman J. EMT, cancer stem cells and drug resistance: an emerging axis of evil in the war on cancer. Oncogene 2010; 29: 4741–4751. 20531305 10.1038/onc.2010.215 PMC3176718

[bib16] 16Ventura A, Luzi L, Pacini S, Baldari CT, Pelicci PG. The p66Shc longevity gene is silenced through epigenetic modifications of an alternative promoter. J Biol Chem 2002; 277: 22370–22376. 11948181 10.1074/jbc.M200280200

[bib17] 17Hardy WR, Li L, Wang Z, Sedy J, Fawcett J, Frank E et al. Combinatorial ShcA docking interactions support diversity in tissue morphogenesis. Science 2007; 317: 251–256. 17626887 10.1126/science.1140114 PMC2575375

[bib18] 18Migliaccio E, Mele S, Salcini AE, Pelicci G, Lai KM, Superti-Furga G et al. Opposite effects of the p52shc/p46shc and p66shc splicing isoforms on the EGF receptor-MAP kinase-fos signalling pathway. EMBO J 1997; 16: 706–716. 9049300 10.1093/emboj/16.4.706 PMC1169672

[bib19] 19Migliaccio E, Giorgio M, Mele S, Pelicci G, Reboldi P, Pandolfi PP et al. The p66shc adaptor protein controls oxidative stress response and life span in mammals. Nature 1999; 402: 309–313. 10580504 10.1038/46311

[bib20] 20Ma Z, Myers DP, Wu RF, Nwariaku FE, Terada LS. p66Shc mediates anoikis through RhoA. J Cell Biol 2007; 179: 23–31. 17908916 10.1083/jcb.200706097 PMC2064727

[bib21] 21Du W, Jiang Y, Zheng Z, Zhang Z, Chen N, Ma Z et al. Feedback loop between p66(Shc) and Nrf2 promotes lung cancer progression. Cancer Lett 2013; 337: 58–65. 23689140 10.1016/j.canlet.2013.05.016

[bib22] 22Ma Z, Liu Z, Wu RF, Terada LS. p66(Shc) restrains Ras hyperactivation and suppresses metastatic behavior. Oncogene 2010; 29: 5559–5567. 20676142 10.1038/onc.2010.326 PMC3045677

[bib23] 23Li X, Xu Z, Du W, Zhang Z, Wei Y, Wang H et al. Aiolos promotes anchorage independence by silencing p66Shc transcription in cancer cells. Cancer Cell 2014; 25: 575–589. 24823637 10.1016/j.ccr.2014.03.020 PMC4070880

[bib24] 24Frisch SM, Schaller M, Cieply B. Mechanisms that link the oncogenic epithelial-mesenchymal transition to suppression of anoikis. J Cell Sci 2013; 126: 21–29. 23516327 10.1242/jcs.120907 PMC3603508

[bib25] 25Zavadil J, Haley J, Kalluri R, Muthuswamy SK, Thompson E. Epithelial-mesenchymal transition. Cancer Res 2008; 68: 9574–9577. 19047131 10.1158/0008-5472.CAN-08-2316

[bib26] 26Smit MA, Peeper DS. Zeb1 is required for TrkB-induced epithelial-mesenchymal transition, anoikis resistance and metastasis. Oncogene 2011; 30: 3735–3744. 21478908 10.1038/onc.2011.96

[bib27] 27Takai E, Tsukimoto M, Kojima S. TGF-beta1 downregulates COX-2 expression leading to decrease of PGE2 production in human lung cancer A549 cells, which is involved in fibrotic response to TGF-beta1. PLoS One 2013; 8: e76346. 24098479 10.1371/journal.pone.0076346 PMC3788736

[bib28] 28Zhang J, Tian XJ, Zhang H, Teng Y, Li R, Bai F et al. TGF-beta-induced epithelial-to-mesenchymal transition proceeds through stepwise activation of multiple feedback loops. Sci Signal 2014; 7: ra91. 25270257 10.1126/scisignal.2005304

[bib29] 29Risolino M, Mandia N, Iavarone F, Dardaei L, Longobardi E, Fernandez S et al. Transcription factor PREP1 induces EMT and metastasis by controlling the TGF-beta-SMAD3 pathway in non-small cell lung adenocarcinoma. Proc Natl Acad Sci USA 2014; 111: E3775–E3784. 25157139 10.1073/pnas.1407074111 PMC4246949

[bib30] 30Zheng Z, Yang J, Zhao D, Gao D, Yan X, Yao Z et al. Downregulated adaptor protein p66(Shc) mitigates autophagy process by low nutrient and enhances apoptotic resistance in human lung adenocarcinoma A549 cells. FEBS J 2013; 280: 4522–4530. 23815759 10.1111/febs.12416

[bib31] 31Shah PP, Lockwood WW, Saurabh K, Kurlawala Z, Shannon SP, Waigel S et al. Ubiquilin1 represses migration and epithelial-to-mesenchymal transition of human non-small cell lung cancer cells. Oncogene 2014; 21: 97. 10.1038/onc.2014.97 PMC4205225 24747970

[bib32] 32Drake LE, Macleod KF. Tumour suppressor gene function in carcinoma-associated fibroblasts: from tumour cells via EMT and back again? J Pathol 2014; 232: 283–288. 24254977 10.1002/path.4298 PMC6664431

[bib33] 33Thiery JP, Acloque H, Huang RY, Nieto MA. Epithelial-mesenchymal transitions in development and disease. Cell 2009; 139: 871–890. 19945376 10.1016/j.cell.2009.11.007

[bib34] 34McClatchey AI, Yap AS. Contact inhibition (of proliferation) redux. Curr Opin Cell Biol 2012; 24: 685–694. 22835462 10.1016/j.ceb.2012.06.009

[bib35] 35Puisieux A, Brabletz T, Caramel J. Oncogenic roles of EMT-inducing transcription factors. Nat Cell Biol 2014; 16: 488–494. 24875735 10.1038/ncb2976

[bib36] 36Varelas X, Samavarchi-Tehrani P, Narimatsu M, Weiss A, Cockburn K, Larsen BG et al. The Crumbs complex couples cell density sensing to Hippo-dependent control of the TGF-beta-SMAD pathway. Dev Cell 2010; 19: 831–844. 21145499 10.1016/j.devcel.2010.11.012

[bib37] 37Wang Y, Shi J, Chai K, Ying X, Zhou BP. The role of Snail in EMT and tumorigenesis. Curr Cancer Drug Targets 2013; 13: 963–972. 24168186 10.2174/15680096113136660102 PMC4004763

[bib38] 38Meng X, Kong DH, Li N, Zong ZH, Liu BQ, Du ZX et al. Knockdown of BAG3 induces epithelial-mesenchymal transition in thyroid cancer cells through ZEB1 activation. Cell Death Dis 2014; 5: e1092. 24577090 10.1038/cddis.2014.32 PMC3944249

[bib39] 39Mani SA, Guo W, Liao MJ, Eaton EN, Ayyanan A, Zhou AY et al. The epithelial-mesenchymal transition generates cells with properties of stem cells. Cell 2008; 133: 704–715. 18485877 10.1016/j.cell.2008.03.027 PMC2728032

[bib40] 40Chaffer CL, Marjanovic ND, Lee T, Bell G, Kleer CG, Reinhardt F et al. Poised chromatin at the ZEB1 promoter enables breast cancer cell plasticity and enhances tumorigenicity. Cell 2013; 154: 61–74. 23827675 10.1016/j.cell.2013.06.005 PMC4015106

[bib41] 41Magenta A, Cencioni C, Fasanaro P, Zaccagnini G, Greco S, Sarra-Ferraris G et al. miR-200c is upregulated by oxidative stress and induces endothelial cell apoptosis and senescence via ZEB1 inhibition. Cell Death Differ 2011; 18: 1628–1639. 21527937 10.1038/cdd.2011.42 PMC3172120

[bib42] 42Afanas'ev I. Reactive oxygen species and age-related genes p66shc, Sirtuin, FOX03 and Klotho in senescence. Oxid Med Cell Longev 2010; 3: 77–85. 20716932 10.4161/oxim.3.2.2 PMC2952092

[bib43] 43Reddig PJ, Juliano RL. Clinging to life: cell to matrix adhesion and cell survival. Cancer Metastasis Rev 2005; 24: 425–439. 16258730 10.1007/s10555-005-5134-3

[bib44] 44Frisch SM, Screaton RA. Anoikis mechanisms. Curr Opin Cell Biol 2001; 13: 555–562. 11544023 10.1016/s0955-0674(00)00251-9

[bib45] 45Simpson CD, Anyiwe K, Schimmer AD. Anoikis resistance and tumor metastasis. Cancer Lett 2008; 272: 177–185. 18579285 10.1016/j.canlet.2008.05.029

[bib46] 46Debnath J. p66(Shc) and Ras: controlling anoikis from the inside-out. Oncogene 2010; 29: 5556–5558. 20711240 10.1038/onc.2010.347

[bib47] 47Arima Y, Inoue Y, Shibata T, Hayashi H, Nagano O, Saya H et al. Rb depletion results in deregulation of E-cadherin and induction of cellular phenotypic changes that are characteristic of the epithelial-to-mesenchymal transition. Cancer Res 2008; 68: 5104–5112. 18593909 10.1158/0008-5472.CAN-07-5680

[bib48] 48Ramirez RD, Sheridan S, Girard L, Sato M, Kim Y, Pollack J et al. Immortalization of human bronchial epithelial cells in the absence of viral oncoproteins. Cancer Res 2004; 64: 9027–9034. 15604268 10.1158/0008-5472.CAN-04-3703

[bib49] 49Liu Z, Garrard WT. Long-range interactions between three transcriptional enhancers, active Vkappa gene promoters, and a 3' boundary sequence spanning 46 kilobases. Mol Cell Biol 2005; 25: 3220–3231. 15798207 10.1128/MCB.25.8.3220-3231.2005 PMC1069589

