# Supplementary figures and images for: Negative feedback loop between p66Shc and ZEB1 regulates fibrotic EMT response in lung cancer cells
Source: Cell Death Dis. 2015 Apr 2;6(4):e1708–. doi: 10.1038/cddis.2015.74 (PMC4650543; doi:10.1038/cddis.2015.74)

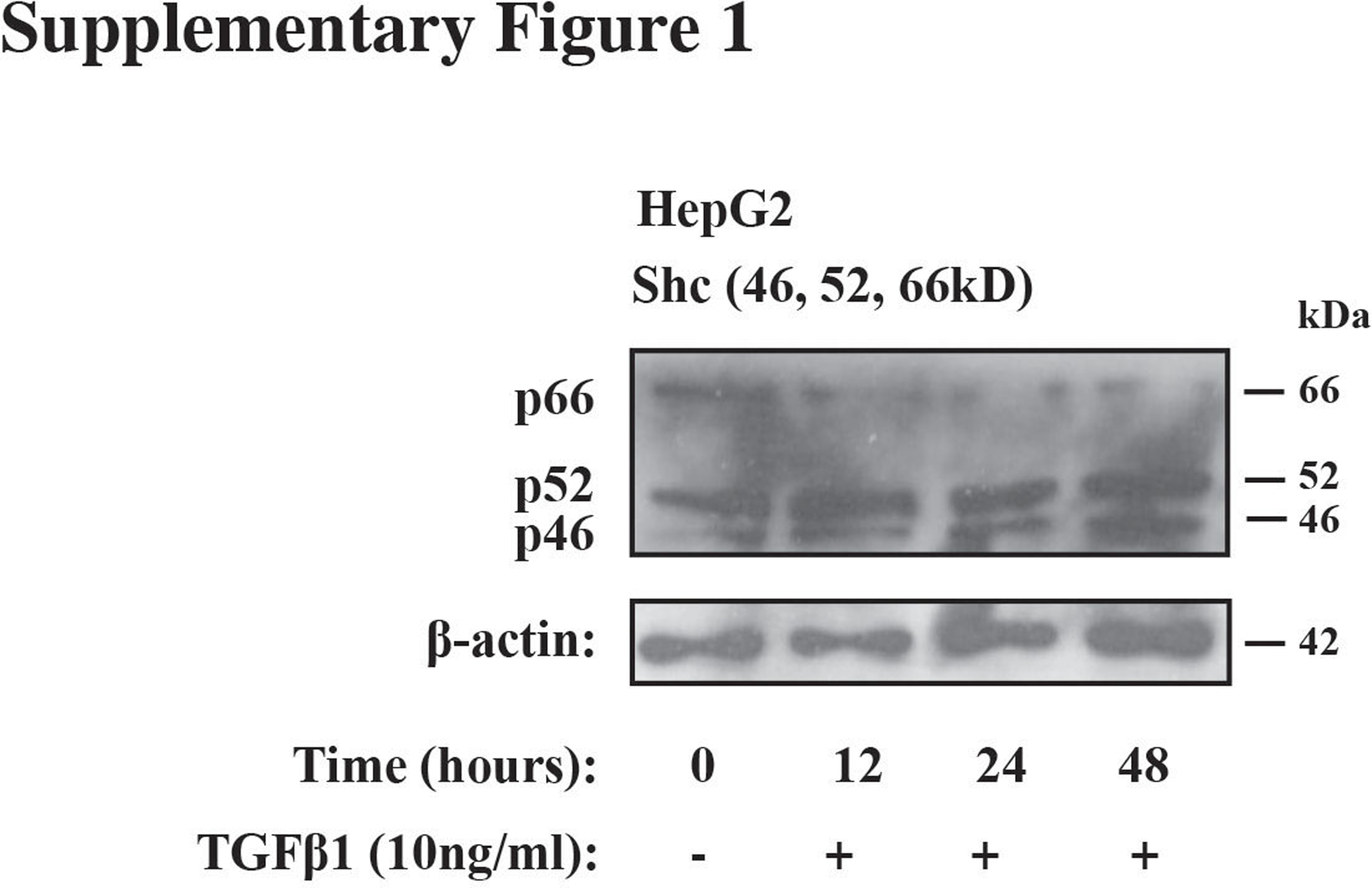

Supplement: Supplementary Figure 1 [file cddis201574x1.tif]

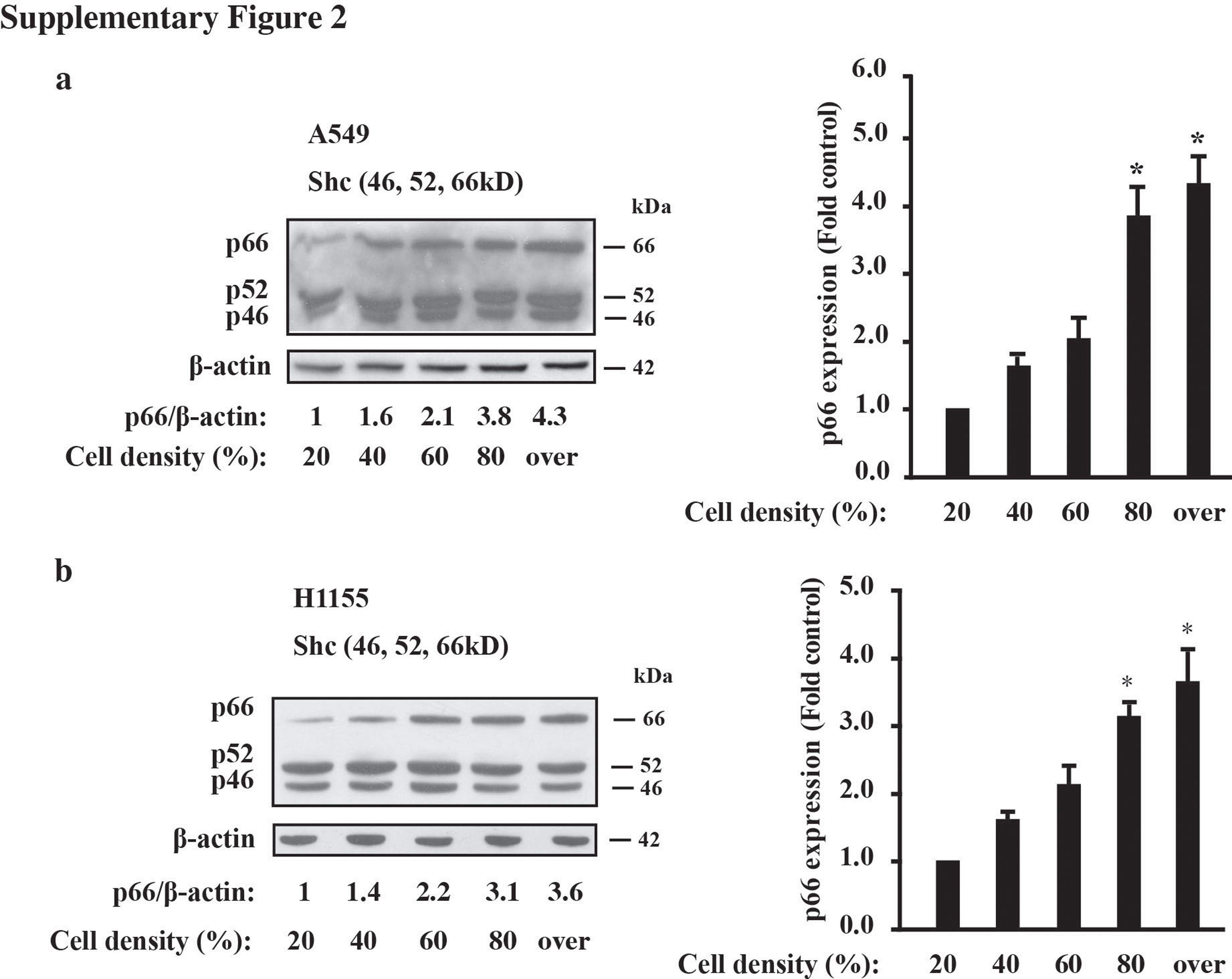

Supplement: Supplementary Figure 2 [file cddis201574x2.tif]

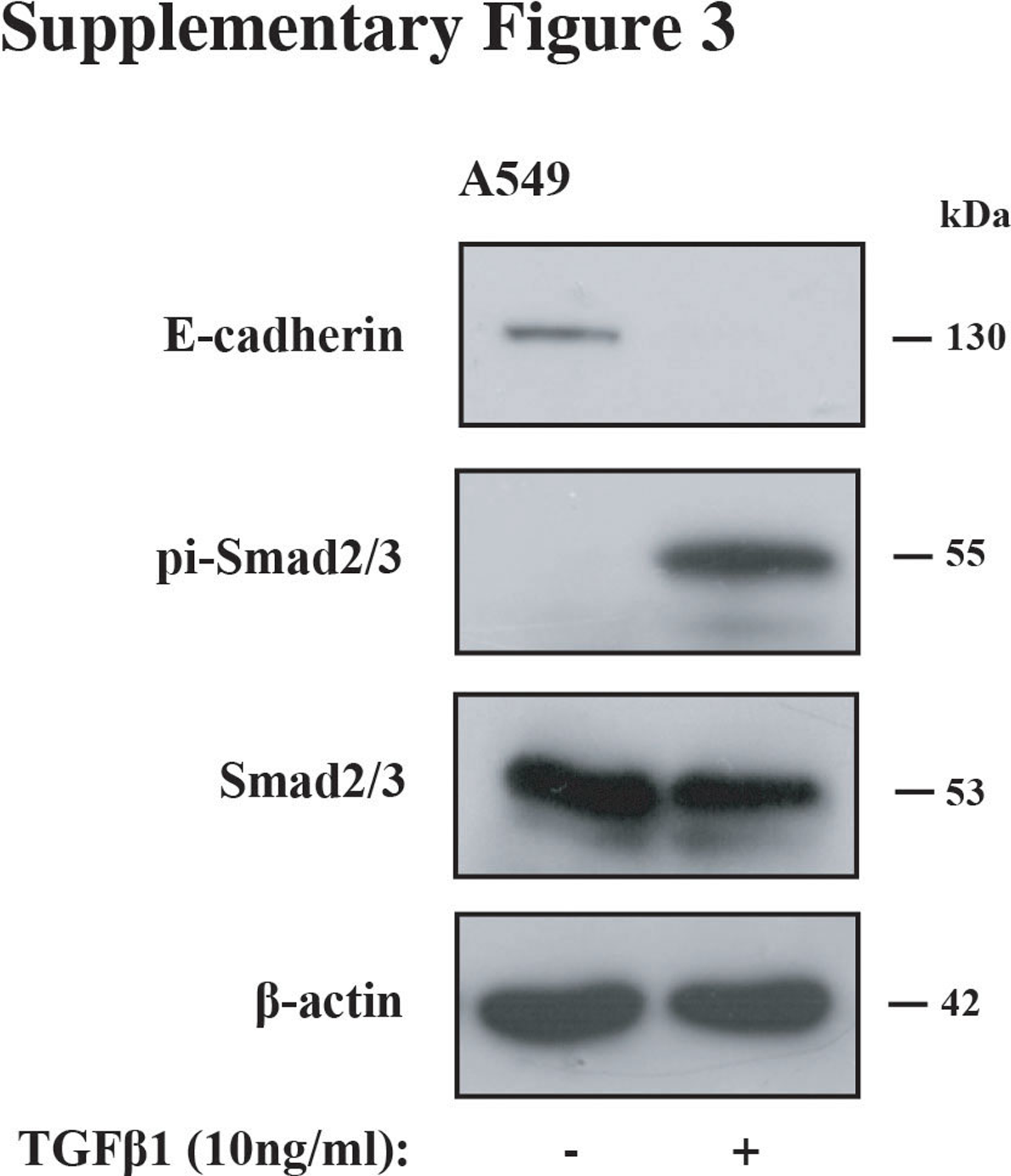

Supplement: Supplementary Figure 3 [file cddis201574x3.tif]

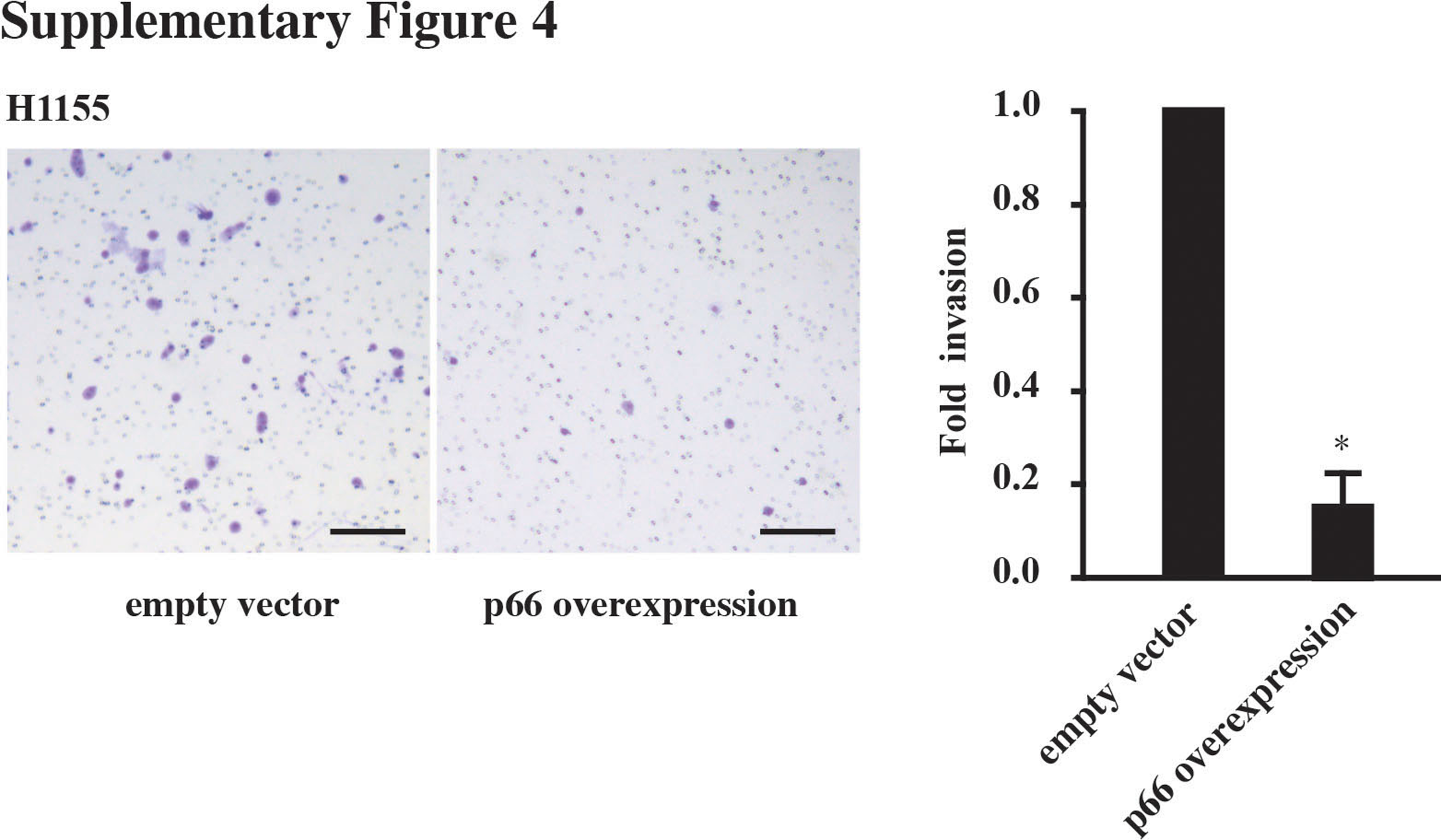

Supplement: Supplementary Figure 4 [file cddis201574x4.tif]
