# Supplementary material for: Negative feedback loop between p66Shc and ZEB1 regulates fibrotic EMT response in lung cancer cells
Source: Cell Death Dis. 2015 Apr 2;6(4):e1708–. doi: 10.1038/cddis.2015.74 (PMC4650543; doi:10.1038/cddis.2015.74)
Supplement: Supplementary Figure Legends [file cddis201574x5.doc]

**Supplementary Figure Legends**

**Supplementary Figure 1** Immunoblot analysis of Shc1 in HepG2 cells with TGFβ1 treatment at the indicated time, with β-actin as a loading control. Results shown are representative of three independent blots.

**Supplementary Figure 2** Immunoblot analysis of Shc1 in A549 (a) and H1155 (b) cells at the indicated cell density as performed in Figure 1d.

**Supplementary Figure 3** A549 cells were treated with TGFβ1 for 12 h and whole cell lysates were subjected to immunoblot analysis with antibodies as indicated. Membrane was stripped and reprobed with β-actin as an equal protein loading control.

**Supplementary Figure 4** Boyden chamber assay for H1155 cells transduced with empty vector or overexpressing p66^Shc^ as described in Figure 2a. Scale bars, 100 μm. Quantification of invasion change shown in right panel. **p* < 0.01 as compared with the cells treated with empty vector alone.
