# Supplementary material for: Negative feedback loop between p66Shc and ZEB1 regulates fibrotic EMT response in lung cancer cells
Source: Cell Death Dis. 2015 Apr 2;6(4):e1708–. doi: 10.1038/cddis.2015.74 (PMC4650543; doi:10.1038/cddis.2015.74)
Supplement: Supplementary Tables [file cddis201574x6.doc]

**Supplementary Table 1: Primer sequences**

| **Primers used for amplification of ZEB1**  *ZEB1-ORF-F…………………… 5’CGG GAT CCG CCA CCa tgG ACT ACA AGG ACG ACG ATG ACA AGa aag tta caa att ata ata ctg 3’*  *ZEB1-ORF-R……………………. 5’ CGG GAT CCt tag gct tca ttt gtc ttt tct tca gac act tgc tc 3’*  **Primers for construction of luciferase reporter assay of p66^Shc^ promoter**  *-3500-+101…………………….… 5’ GGC CCT GGT GAG TCT TGA CT 3’*  *reverse primer…………………… 5’ AGA AGT CCT GGG GAG GGA GA 3’*  **Targeting sequence for shRNA**  *p66^Shc^shRNA(1)………….…….… 5’ GAA TGA GTC TCT GTC ATC G 3’*  *p66^Shc^shRNA(2)…………….….… 5’ AGG AAG GGC agC tGA TGA t 3’*  *luciferase shRNA………………… 5’ CTT ACG CTG AGT ACT TCG A 3’*  **Primers used for Semi-Quantitative RT-PCR**  *GAPDH-SemiQ-F………………… 5’ GTC AAC GGA TTT GGT CGT ATT 3’*  *GAPDH-SemiQ-R………………… 5’ AGT CTT CTG GGT GGC AGT GAT 3’*  *ZEB1-SemiQ-F…………………… 5’ CAG GAA AGG AAG GGC AAG AA 3’*  *ZEB1-SemiQ-R…………………… 5’ CTG CAC AGG GAG CAA CTA AA 3’*  *p66^Shc^-SemiQ-F…………………… 5’ aat gag tct ctg tca tcg ctg gag 3’*  *p66^Shc^-SemiQ-R…………………… 5’ ggc gat gat ctg ttt gca gtc tgc gg 3’*  **Primers used for Real-Time PCR**  *GAPDH-F…………….………… 5’ GGT GGT CTC CTC TGA CTT CAA CA 3’*  *GAPDH-R…………….………… 5’ GTT GCT GTA GCC AAA TTC GTT GT 3’*  *p66^Shc^-F……………….………… 5’ aat gag tct ctg tca tcg ctg gag 3’*  *p66^Shc^-R……………….………… 5’ gag cac agg gta gtg gga cta t 3’*  *CDH1-F………………….……… 5’ ACA CTG CCA ACT GGC TGG AGA TTA 3’*  *CDH1-R………………….……… 5’ TGA TTA GGG CTG TGT ACG TGC TGT 3’*  *CTNNBIP1-F…………………… 5’ GTT GAG CAC CTG TTT GCC TGA AGT 3’*  *CTNNBIP1-R…………………… 5’ TCA GGT TTG ATC CCA TCT TCC GCA 3’*  *KRT20-F………………………… 5’ TGA AGT CAT GGC CCA GAA GAA CCT 3’*  *KRT20-R………………………… 5’ TGC GTC TCA GCT CCG TTA GTT GAA 3’*  *FN-F…………………..………… 5’ CAG TGG GAG ACC TCG AG AAG 3’*  *FN-R…………………..………… 5’ TCC CTC GGA ACA TCA GAA AC 3’*  *VIM-F…………………………… 5’ GAG AAC TTT GCC GTT GAA GC 3’*  *VIM-R…………………………… 5’ GCT TCC TGT AGG TGG CAA TC 3’*  *CDH2-F………………………… 5’ ACA GTG GCC ACC TAC AAA GG 3’*  *CDH2-R………………………… 5’ CCG AGA TGG GGT TGA TAA TG 3’*  *SNAI1-F………………………… 5’ TTC TCT AGG CCC TGG CTG CTA CAA 3’*  *SNAI1-R………………………… 5’ TCT TGA CAT CTG AGT GGG TCT GGA 3’*  *SNAI2-F………………………… 5’ ACC TTG TGT TTG CAA GAT CTG CGG 3’*  *SNAI2-R………………………… 5’ TGC AAA TGC TCT GTT GCA GTG AGG 3’*  *ZEB1-F…………….…………… 5’ AGC GCT AGC TGC CAA TAA GCA AAC 3’*  *ZEB1-R…………….…………… 5’ TTG GGC GGT GTA GAA TCA GAG TCA 3’*  *ZEB2-F…………….…………… 5’ TTC CTG GGC TAC GAC CAT AC 3’*  *ZEB2-R…………….…………… 5’ TGT GCT CCA TCA AGC AAT TC 3’*  **Primers for ChIP**  *SHC1-1F ................................ 5’ GAG GCG GGA CAC CTC TAC TTT CAA G 3’*  *SHC1-1R ................................ 5’ AGC CAA TGC AGC GCG AGA T 3’*  *SHC1-2F................................. 5’ GTG ACT CAC TCT TTC ACC CCG 3’*  *SHC1-2R ................................ 5’ GTG GGG TCG GAG ACA AAA 3’*  *SHC1-3F ................................ 5’ TTT CGC TCT TAT CGT CCA GG 3’*  *SHC1-3R ................................ 5’ CGC CTG TAA TCC TAA CAC TTA AGG 3’*  *SHC1-4F ................................ 5’ TCC CCC TAA CCA GAG TTC TTG 3’*  *SHC1-4R ................................ 5’ TAG GGG AAA AGC TGT GTG TG 3’*  *SHC1-5F................................. 5’ GAG ACA GGA CAG TGC TTG GC 3’*  *SHC1-5R ................................ 5’ GGA AGA GCA AAG CTG GTG AA 3’*  *SHC1-6F ................................ 5’ TGA GTC AGC ACT GTC CTC ACG ATT 3’*  *SHC1-6R ................................ 5’ AAG GTG GAG CCT CTT CTC TGG CT 3’*  *SHC1-7F................................. 5’ TTC TAA TGC TCA CTC CAG CTT GGC 3’*  *SHC1-7R................................. 5’ GGG AAG GGA TGA GAA AGG TTT AGG 3’* |
| --- |
